# The central role of vascular extracellular matrix and basement membrane remodeling in metabolic syndrome and type 2 diabetes: the matrix preloaded

**DOI:** 10.1186/1475-2840-4-9

**Published:** 2005-06-28

**Authors:** Melvin R Hayden, James R Sowers, Suresh C Tyagi

**Affiliations:** 1 Department of Family and Community Medicine, University of Missouri School of Medicine Columbia, Missouri PO BOX 1140 Lk. Rd. 5–87 Camdenton, Missouri 65020 USA; 2 Department of Internal Medicine, University of Missouri School of Medicine Columbia, Missouri Health Sciences Center, MA410, DC043.00 Columbia, Missouri 65212 USA; 3 Department of Physiology and Biophysics, University of Louisville, School of Medicine 500 South Preston Street University of Louisville Louisville, Kentucky 40292 USA

**Keywords:** Atherosclerosis, Collagen, elastin, proteoglycan, structural glycoprotein, Integrin, oxidative stress, redox stress, MMP, TIMP, remodeling

## Abstract

The vascular endothelial basement membrane and extra cellular matrix is a compilation of different macromolecules organized by physical entanglements, opposing ionic charges, chemical covalent bonding, and cross-linking into a biomechanically active polymer. These matrices provide a gel-like form and scaffolding structure with regional tensile strength provided by collagens, elasticity by elastins, adhesiveness by structural glycoproteins, compressibility by proteoglycans – hyaluronans, and communicability by a family of integrins, which exchanges information between cells and between cells and the extracellular matrix of vascular tissues.

Each component of the extracellular matrix and specifically the capillary basement membrane possesses unique structural properties and interactions with one another, which determine the separate and combined roles in the multiple diabetic complications or diabetic opathies.

Metabolic syndrome, prediabetes, type 2 diabetes mellitus, and their parallel companion (atheroscleropathy) are associated with multiple metabolic toxicities and chronic injurious stimuli. The adaptable quality of a matrix or form genetically preloaded with the necessary information to communicate and respond to an ever-changing environment, which supports the interstitium, capillary and arterial vessel wall is individually examined.

## Background

A matrix may be defined as something within or from which something else originates, develops, or takes form. The extracellular matrix (ECM) is a post-natally developed mesenchyme and provides scaffolding and structural support for cells and organs. Additionally, it is capable of exchanging information with cells and thereby modulates a whole host of processes including development, cell migration, attachment, differentiation, and repair. The repairing aspect of the ECM allows it to play a crucial role in wound healing via its chemotactic, haptotactic, opsonic, and ultimate attachment properties.

**Figure 1:**
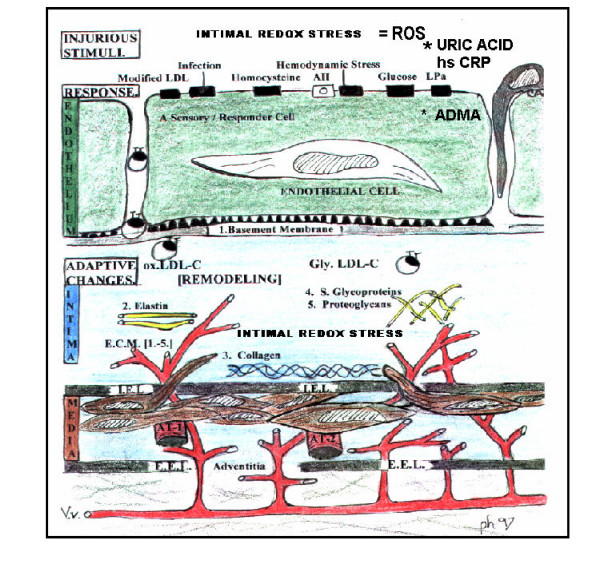
**multiple injurious stimuli to the Endothelium, intima, media, and adventitia. **The endothelial cell is exposed to multiple injurious stimuli consisting of: modified LDL-cholesterol, various infection insults (viral and bacterial), angiotensin II, hemodynamic stress, LPa, glucose, homocysteine, uric acid, Ca++, phosphorus, parathyroid hormone, and intimal redox stress or reactive oxygen species. These multiple injurious stimuli (A-FLIGHT-U) cause a chronic injury and a response to injury with resultant remodeling of the arterial vessel wall and in particular the ECM. In the MetS, prediabetes, and overt T2DM, these stimuli act in concert to result in this detrimental remodeling with structural-functional abnormalities and dysfunction. The endothelium and its BM act as the first line of defense and are therefore the first cell and matrix to be affected with resulting dysfunction and structural changes. MetS, prediabetes, and T2DM undergo an accelerated atherosclerosis we term atheroscleropathy. Oxidation, glycation, glycoxidation, or homocysteinylation must modify LDL-cholesterol for LDL-C to become atherogenic. Multiple injurious stimuli acting alone and synergistically to modify LDL-cholesterol with resultant matrix structural defects accelerating atherogenesis and angiogenesis are observed. Each layer of the arterial vessel wall is eventually affected by these injurious stimuli initially from the lumen outward (inside-out) and later in the process to effect the plaque vulnerability from the outside-in (adventitial layer) by an inducible set of custom delivery vessels called the vasa vasorum.

Metabolic syndrome (MetS) and type 2 diabetes mellitus (T2DM), which are now considered to be of pandemic proportions are conditions associated with multiple metabolic toxicities (table 1) and chronic injurious stimuli (figure 1). When uncontrolled by chronic injurious stimuli, there is chronic activation of these above processes resulting in fibrosis, structural derangement, tissue or organ dysfunction, and ultimate failure as a result of loss of form – structure and function.

**Table 1:** The multiple metabolic toxicities of the A-flight-u Acronym

Multiple injurious stimuli responsible for the production of ROS.	
**A**	Angiotensin II (also induces protein kinase C – β isoform) Amylin (hyperamylinemia) islet amyloid polypeptide toxicity AGEs/AFEs (advanced glycosylation/fructosylation endproducts) Apolipoprotein B Antioxidant reserve compromised Absence of antioxidant network Aging ADMA (Asymmetrical DiMethyl Arginine) Adipose toxicity: **Obesity toxicity – Lipid Triad **(decreased HDL-C, increased triglycerides and small dense LDL-C) Adipocytokine toxicity: Increased TNF alpha, Il-6, TGF beta, PAI-I and the increased hormones resistin, leptin and decreased adiponectin.

**F**	Free fatty acid toxicity: **Obesity toxicity – Lipid Triad**

**L**	Lipotoxicity – Hyperlipidemia – **Obesity toxicity – Lipid Triad **Leptin toxicity

**I**	Insulin toxicity (endogenous hyperinsulinemia-hyperproinsulinemia) IGF-1 – (GH-IGF-I axis) toxicity: This may serve to increase bone metabolism within the media of the AVW Inflammation toxicity

**G**	Glucotoxicity (compounds peripheral insulin resistance) and induces reductive stress through the sorbitol/polyol pathway Pseudohypoxia (increased NADH/NAD ratio)

**H**	Hypertension toxicity Homocysteine toxicity hs-CRP

**T**	Triglyceride toxicity: **Obesity toxicity – Lipid Triad**
**U**	Uric Acid – Xanthine Oxidase toxicity: Uric acid is an antioxidant early in physiological range of atherogenesis and a conditional prooxidant late when elevated through xanthine oxidase enzyme and generation of ROS: thus generating the paradoxical (antioxidant → prooxidant): ***URATE REDOX SHUTTLE*** Endothelial cell dysfunction with eNOS uncoupling, decreased eNO and increased **ROS**

## The Component quintology of the ECM

The ECM consists of the following quintet: basement membrane (BM), collagen, elastin, proteoglycans (glycosaminoglycans – GAGs) and hyaluronan, and structural – adhesive glycoproteins.

### I. Basement membrane (BM): (intimal and capillary)

The BM is important for the physical support and cellular attachment of cells and maintenance of their structural integrity, thus allowing cells to create and maintain their own special environment and provides a filtering – sieving mechanism due to the strong anionic charges of its matrix.

The importance of the ECM and the thickened capillary BM in diabetes was brought to "prime time" attention of diabetologists and researchers in 1968 with the publication of a paper by Siperstein MD and colleagues [1]. During the decade of the 70s others became interested in this phenomenon of matrix expansion within the basement membrane [2-10]. Specifically, Williamson JR and Kilo C were very strong contributors to this exciting area of science and they contributed strongly to the concept that diabetics have "leaky" blood vessels and that glucose was toxic to the endothelial cell and instigated capillary BM thickening [11-29].

Throughout the decades of the 1970's and 1980's; a common vernacular terminology used to describe diabetes was the following:

"Diabetes is a basement membrane disease."

This terminology is infrequently used today even though it is a widely accepted concept (capillary BM thickening is an ultrastructural hallmark in diabetic patients). This review will focus on the importance of the remodeled thickened CBM and update each component of the ECM. Additionally, an attempt will be made to show how the ECM comes "preloaded" with multiple reparative mechanisms to undergo the morphological structural change of remodeling in response to the metabolic and pathobiomolecular mechanisms associated with MetS, prediabetes, and overt T2DM.

The BM is a specialized extracellular matrix, which provides support – cell regulatory and filtering – sieving functions. Endothelial cells and most other epithelial cells are capable of synthesizing their BM.

**Figure 2:**
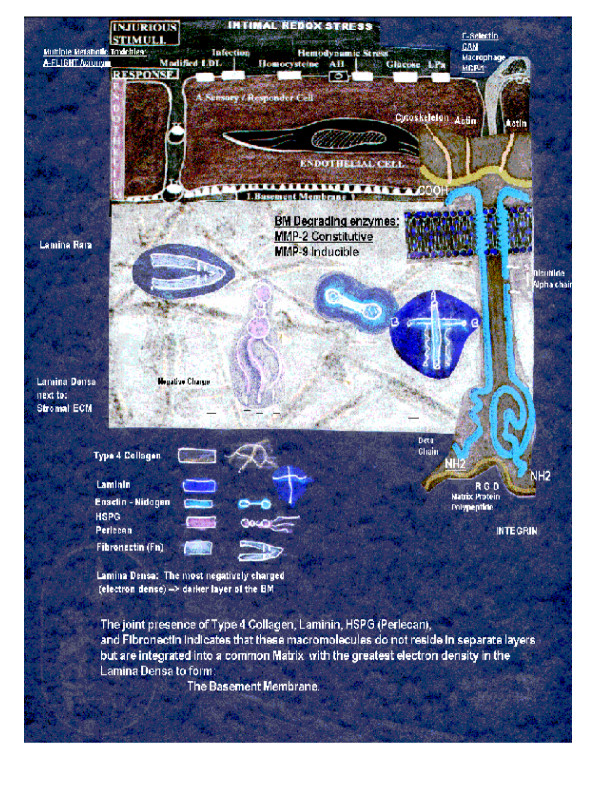
**The basement membrane exploaded. **This image expands – explodes the BM and demonstrates the importance of each of its components that are involved in the expansion and thickening of the BM in MetS, prediabetes, and T2DM. The BM is an integral part of the ECM and plays such an integrating role in the structural – functional changes associated with MetS, prediabetes, and T2DM. An integrin has been placed in this image to verify its important-integrating role in cell-cell, cell-matrix communication. This image demonstrates the existence of a shape and a form to the PAS+, hyaline staining, thickened BM in MetS, prediabetes, and T2DM.

MetS, prediabetes and T2DM are characterized by perturbations of the arterial vasculature, especially the endothelium and capillary BM, which are integrally involved with profound cardiovascular and microvascular complications. The endothelium and its BM are the first line of defense against injurious stimuli at the vascular lumen and capillary bed and are responsible for the regulation of vascular tone, circulation, fluidity, coagulation, inflammatory responses, oxidative stress, and remodeling in response to injurious stimuli (figure 1, 2).

The interactions of the endothelial cell, its endothelial capillary BM, and their associated ECM become major players in the developing complications of MetS, prediabetes, and T2DM and are the central issue of this review. Proposed mechanisms of increased ECM accumulation in the BM (table 2) are rooted in multiple metabolic toxicities and reactive oxygen species (ROS) associated with MetS and T2DM and have a multifactorial pathogenesis.

**Table 2:** Observations and proposed mechanisms of increased capillary bm thickening with appliations to the myocardial, intima, islet, neuronal unit, Endothelial, renal, retinal and skeletal capillary basement membranes (Increased synthesis and decreased degradation tips the balance to accumulation)

**Observations**	**Proposed mechanisms**
Glucotoxicity: IGT postprandial, IFG, and overt T2DM	Protein Kinase C (PKC) activation. Altered integrin expression of podocyte and pericyte: (podocyte-pericyte loss)
Increased Synthesis Of: Type Iv Collagen	TGF beta, VEGF, and possibly PDGF beta 1. All associated with PKC activation and induction of growth hormones – factors from glucotoxicity and → ROS.
Increased Maintenance of: type IV Collagen AGE cross-linking of type IV collagen. **AGE – RAGE connection.**	Increased resistance to protease (MMP) degradation, allowing type IV collagen to accumulate.
Decreased Degradation.	Decreased MMP-2 MMP-3
Decreased Degradation.	Increased expression of TIMP-2
Increased ROS. In general, ROS promotes ECM fibrosis under the influence of chronic injurious stimuli and is associated with the chronic inflammatory state.	ROS increases all aspects of type IV accumulation: Glucotoxicity → PKC activation, AGE cross-linking activation, Decreased eNOS and eNO activity resulting in increased MMP activation.*

*Comment: It seems whenever there is robust MMP activation the result is robust newly synthesized collagen, which is more susceptible to ROS oxidation and accumulation. In general, it is more difficult to degrade newly synthesized – oxidized collagen than non-oxidized collagen. Other observations include an association of decreased eNO and increased activation of MMPs and in a like manner when eNO is normal or elevated MMP activity is suppressed. This comment points to the importance of a healthy eNOS and eNO generating endothelium in order for the ECM to maintain homeostasis.

Endothelial nitric oxide synthase (eNOS) – Endothelial nitric oxide (eNO)

Accumulation of BM material in renal tubular cells, the endothelial capillary beds of the renal, retinal, neuronal unit, myocardial and skeletal muscle, and the arterial vasculature itself are at the very core of these disease processes and diabetic complications. Even though these remodeled BMs appear thickened on microscopic examination, they lose their filtering – sieving (permselectivity) function and become dysfunctional due to a leakiness of larger proteins (such as albumin and lipoproteins into the intima and sub-capillary interstitium) and inflammatory cells [1-29]. The remodeling of the endothelial BM may also make the endothelium more prone to erosion and thrombosis in patients with metabolic abnormalities.

**II. *Collagen ***is the most abundant protein in humans and provides the framework for all multi-cellular organisms. There are characteristic triplet repeats of amino acids in the collagen molecule consisting of glycine XY, which results in glycine being present in every third amino acid. The collagen molecule is formed by three polypeptide chains, which intertwine to form triple helical rope-like collagen fibrils. These fibrils are cross-linked by hydroxyl groups between alpha chains (a major contributor to their tensile strength) to form the collagen fiber and these fibers, in turn, form collagen bundles. Gaps in the collagen fibril give the cross-banding appearance of types I and II collagen fibers at a characteristic length of 67 nm when viewed by electron microscopy. In type III collagen there is a structurally beaded appearance instead of the characteristic cross-banding appearance observed in types I and II collagen.

The physical and tensile strength of collagens are typified by collagen type I (having the tensile strength of steel), which predominates in bones, tendons, skin, and mature scars, while type II collagen is thinner and predominates in cartilage, vitreous humor and nucleus pulposus. Type III collagen is found in organs requiring more plasticity such as blood vessels, heart, gastrointestinal tract, uterus, and the dermis.

Types I, II, and III collagen are the fibrillar – interstitial collagens and are the most abundant collagen types. They are important in diabetic remodeling fibrosis within the myocardium (cardiomyopathy), the tubulo-interstitium of the kidney (nephropathy-interstitiopahy), the intima (intimopathy) in atheroscleropathy, dermopathy, interstitial changes within the retina (retinopathy), and possibly the neuronal unit of neuropathy. In contrast, collagens IV, V, and VI are non-fibrillar or amorphous and are found in BMs and interstitial tissue.

Historically, BMs have been shown to be highly insoluble and possess a distinct stability against mechanical forces. These findings are correlated with the presence of large amounts of a collagenous protein, which differ from the fiber-forming fibrillar collagens type I through III and thus the term type IV collagen has emerged.

One very unique feature of type IV collagen is the presence of seven to eight cysteine residues, which are involved in intra and intermolecular disulfide bonds, which aid in the stabilization of this polymer. This presence of cysteine in type IV collagen is in contrast to mature fibrillar collagens type I, II, and III, as they lack a cysteine moiety.

Type IV collagen is found exclusively in BM and it does not form individual fibers with electron microscopic cross-banding like the other collagens but instead forms an amorphous polygonal matrix, which is associated with laminin and other matrix macromolecules to form the unique BM matrix (figure 2).

Turnover of type IV collagen is known to be very slow unless there are inciting injurious stimuli that activate the specific BM degrading enzymes: matrix metalloproteinase(s) MMP-2 (constitutive) and MMP-9 (inducible) [31-34].

**III. *Elastin ***is known to provide support and elasticity. This elasticity is important for many tissues and organs such as the blood vessels, heart, skin, lung, and uterus. Elastin is a 70-kd glycoprotein and constitutes the central core of elastic fibers. It is similar to collagen, in that it is rich in glycine and proline, but unlike collagen, it contains almost no hydroxylated amino acids. It is cross linked, but unlike most other proteins it does not form definite folds but rather oscillates between different states to form random coils. It is this cross-linked, random-coiled structure of elastin that determines the capacity of the elastic network to stretch and recoil. Fibrillin microfibrils (a unique glycoprotein microfibril) are stiffer reinforcing fibers in compliant tissues and have been recently identified to be associated with elastic fibers [35].

Elastin is not felt to be a primary component of the capillary BM and it is interesting to note that the capillary tuft of the glomerulus was found to be devoid of elastin and present only in the mesangial stalk and afferent and efferent arterioles [36]. This may be one of the reasons that the capillary BM of the glomerular tuft undergoes remodeling expansion and results in a thickening of its BM when exposed to increased volume or increased pressure (intraglomerular hypertension), which occurs in the MetS and early in the natural history of T2DM.

Elastin provides an elastic molecular recoil phenomenon to the ECM and this is why there is a distinct internal and external elastic lamina on either side of the medial vascular smooth muscle layer of the arterial vessel wall (figure 1).

### IV. Proteoglycan(s) (PG) – (glycosaminoglycans – GAGs) and Hhyaluronan

PG and hyaluronan are ubiquitous and found within the intima. They are synthesized primarily by the vascular smooth muscle cell (VSMC), other cells of mesenchymal origin, and in BM by the endothelium.

PG consist of a core protein(s) covalently linked to one or more highly sulfated polysaccharide chains termed glycosaminoglycans (GAGs). These molecules are highly diverse with multiple combinations of core proteins and polysaccharide chains. Examples are: heparan sulfate proteoglycans (HSPG), chondroitin sulfate proteoglycans, keratan sulfate proteoglycans, and dermatan sulfate proteoglycans (table 4).

**Table 4:** A representation of proteoglycans I – IV present in vascular ECM

***Family (location)***	***Common name***	***Function***
**I. Large (interstitial)**	**Versican (CSPG)**	Compression resilience. Similar to Hyaluronan
**II. Small (leucine-rich)**	**Decorin** **Biglycan** **Lumican**	Collagen organization.
**III. Basement membrane Recently discovered → ** In diabetic renal BM the CSPG Bamacan may be substituted for the HSPG Perlecan	**Perlecan (HSPG)** **Bamacan (CSPG) (galactosaminoglycan chains)** **Agrin (HSPG)**	Anionic filtration barrier Binds growth factors Neural tight junctions (Blood Brain Barrier) and in renal BM
**IV. Cell surface **(Plasma membrane). **I.- IV.** **Present in vascular ECM The vascular SMC is the principal source for these vascular proteoglycans.**	**Syndecan-1** **Fibroglycan** **N-Syndecan (HSPG)** **Ryudocan** **Glypican (HSPG)**	ECM receptors, growth factor receptors. Binds coagulant enzymes, cytokines, and lipases.
**V. Cerebral Proteoglycans** **Others:**	***Cerebrocan*** ***Neurocan (CSPG)*** ***Phosphacan (CSPG)*** ***Brevican *(CSPG)** _______ Aggrecan Betaglycan	Prominent in cartilage

They have multiple roles in regulating matrix structure such as cell growth and differentiation and permeability. They are highly sulfated and possess an anionic or negative charge, which makes them ideal to play the important role of selective filtering in the BM, especially in the renal glomerulus.

In this review we are mainly focused on the heparan sulfate proteoglycans (HSPG) (specifically perelecan of the capillary BM) and their role in BM filtering function due to the anionic charge provided by sulfation of the polysaccharide chains. In diabetes there is known to be decreased levels of perlecan in the glomerular BM and in the BM of endothelial, epithelial, and renal tubular cells, which would allow for the loss of an effective filtering function and these observations play a central role in the development of diabetic micro and macroalbuminuria.

This loss of filtering function is associated with the loss of perlecan and is also associated with the increased permeability of the microvessels throughout the vasculature affecting most of the diabetic complications and vasculopathies [37]. There are undoubtedly multiple causes for this decrease in perlecan and the multiple metabolic A-FLIGHT-U toxicities are related to this decrease in perlecan within the BM (table 1, 4). For example: Elevated levels of LDL-cholesterol and oxidized LDL-cholesterol, as well as, lysolecithin decrease not only perlecan core protein synthesis but also enhance heparan sulfate degradation by stimulating endothelial secretion of heparanase. ApoE and apoE-HDL, in contrast, increase perlecan core protein as well as sulfation of heparan sulfate [38]. Additionally non-esterified fatty acid or free fatty acid elevation has been shown to alter PG synthesis within the intima and contribute to LDL-cholesterol retention as well as allowing for increased permeability through an alteration in PG synthesis [39].

Recently it has been suggested that the PGs and the structural – adhesive glycoproteins and their associated glycosaminoglycans (GAG) side chains form a unit, which has been termed the glycocalyx. This unit may serve as a mechanosensor for both endothelial nitric oxide and prostacyclin responses of the endothelium to shear stress [40].

Syndecans form the largest group of HSPG on the endothelial surface and are set apart by being the only HSPG that penetrates the cytoplasm, allowing for an interaction with the cytosolic cytoskeleton (enabling an "outside in" mechanosensing capability). Glypicans form the second most common HSPG group, and have structural similarities to syndecan, typically differing only in the number of GAG attachment sites while perlecan remains in the basement membrane as discussed earlier [41].

This allows the glycocalyx to sense changes in shear stress from the outside and communicate with the G-protein receptors, including those that form a cytoplasmic bond with endothelial nitric oxide synthase and cytoskeletal elements like actin that can transduce physical forces throughout the cell to affect cellular function [42].

The nomenclature regarding PGs will undoubtedly undergo changes in the near future as an attempt to relate the specific PGs to their genomic origins. An attempt to aid in the classification of various PGs relating to the vascular ECM is presented at this point in time with some names of PGs in other tissues also being represented (table 4). Since there are present an unlimited number of possible interactions and combinations of the various PGs, there will undoubtedly become a new and improved nomenclature in the near future.

Other PGs such as versican, biglycan, and decorin accumulate in developing atherosclerotic and restenotic lesions. They contribute to plaque burden and influence cellular and extracellular events associated with the pathogenesis of vascular lesions, such as migration and proliferation, lipid metabolism and retention, and thrombosis.

Additionally, PGs also interact with other components of the ECM and contribute to their ability to regulate biomechanical properties of vascular lesions and even the ability of plaques to resist rupture.

**IV. *Hyaluronan (HA) ***is a huge molecule consisting of disaccharides stretched end-to-end, while lacking a core protein. It binds large amounts of water and forms a viscous hydrated gel, which gives the ECM turgor and allows it to resist compressive forces. Because of this unique ability it is found in abundance in cartilage of joints as it provides resilience and lubrication. It serves as a ligand for core proteins and is often a backbone for large proteoglycan complexes. It facilitates cell migration and inhibits cell-cell adhesion. It is synthesized primarily by the VSMC and is important in the development and progression of atherosclerosis, as well as, the process of post angioplasty restenosis. It communicates primarily through the integrin CD44 and is associated with angiogenesis. Hyaluronan is increased along with VSMC in atherosclerotic plaque erosion and is decreased in the vulnerable thin-cap atheroma associated with plaque rupture.

### V. Structural – adhesive glypoproteins

Fibronectin, a large glycoprotein (approximately 450 kDa), is one of the first structural macromolecules to be deposited during embryonic development. It forms a primitive matrix that allows the initial organization to be replaced by the definitive, organ-specific matrix. The embryologic role of tissue fibronectin as the initial undifferentiated matrix is recapitulated in the early phases of injurious wound healing.

Fibronectin is a multifunctional adhesive protein whose primary function is to attach cells to a variety of matrices. Structurally, it consists of two polypeptide chains held together by two disulfide bonds. In addition to providing structural support it is associated with cell surfaces, pericellular matrices, and BMs. It is synthesized primarily by the cells of mesenchymal origin such as fibroblasts, monocytes, and endothelial cells.

Fibronectin binds to collagen, fibrin, and proteoglycans via specific domains and to cells via receptors that recognize the specific amino acid tripeptide RGD sequence (arginine-glycine-aspartic acid). This RGD integrin-binding motif is felt to be important for the haptotaxsis migration of cells within the ECM. A good example is the migration of capillary endothelial cells within the ECM during the process of capillary angiogenesis. Laminin (820 kDa) is the most abundant glycoprotein in BMs. This structural- adhesive glycoprotein binds to cells, heparan sulfate proteoglycans, and type IV collagen. Laminin is a hetero-trimeric polypeptide and appears as a cross-like structure with a single central polypeptide A chain and two flanking polypeptide B chains, which turns outward at right angles. This adhesive glycoprotein is felt to be important in cellular alignment (figure 2) [40-44].

Enactin and nidogen are different names for the same macromolecule. It is a dumbbell shaped structural – adhesive glycoprotein of ~150 kDa consisting of a 1217 amino acid residue. It has binding properties to both laminin and type IV collagen and thus can act as an adhesive bridge and is important in assembling these two major BM proteins [43,44] (figure 2). In addition to its role of assembling type IV collagen and laminin, Lebel SP *et al*. has been able to demonstrate it has permselective properties through an alteration of the anionic charges, as well as, promoting a morphological thickening of the BM as demonstrated in the enactin null transgenic mouse model [45].

Thus, these initially reparative mechanisms (the remodeling of the ECM), when stimulated by the chronic injurious stimuli associated with MetS, prediabetes, and T2DM result in devastating structural – functional complications.

## Cellular integrins and ECM ligand binding

As mentioned previously the ECM comes genetically preloaded with a vast amount of information in addition to its scaffolding and structural supporting capabilities. Therefore, it is imperative that a brief discussion of the mechanisms allowing this communication of information be discussed.

**Figure 3:**
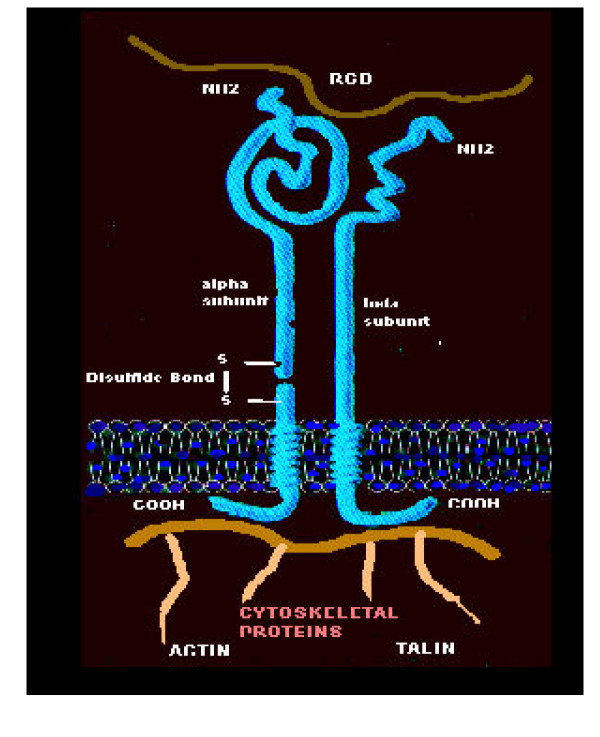
**The integrins. **This image portrays the integrin family of transmembrane molecules (receptors), which interact with the molecules of the ECM (ligands) and ligands associated with other cellular elements. Integrins are heterodimers, consisting of an alpha unit on the left made up of two disulfide bonded polypeptide chains. The beta unit on the right consists of a single polypeptide chain. Integrins bind to matrix ligand binding sties, which are specific amino acid sequences (usually 3–8) and at the top of this image the RGD (arginine, glycine, and aspartyl amino acids) matrix ligand – binding site is demonstrated. There are three domains: the extracellular domain – the transmembrane domain – the cytosolic domain, which interact with the cytosolic cytoskeletal proteins: Actin and Talin. These special transmembrane molecules allow for the "outside in" and "inside out" communication between cells and the ECM. Glucotoxicity affects integrin function through decreased perlecan within BMs and this may increase the susceptibility of endothelial cell dysfunction and demise (apoptosis), allowing for endothelial cell erosion and loss of endothelial cell stability upon its BM increasing the possibility of plaque erosion – rupture and thrombosis.

The integrins comprise a molecular family of cell surface receptors and communicators that bind to the components of the ECM including collagen, laminin, and fibronectin. This interaction between the ECM and cellular integrins allow for a bi-directional (outside in and inside out) exchange of information facilitating a cell matrix communication (figure 3). A specific matrix ligand-binding site of the ECM proteins, consisting of a tripeptide sequence known as the RGD, binds to the integrins on the cell surface.

A simple analogy to the personal computer can be made, as integrins are the modern day computers of the cell, which span the plasma membrane with an extracellular domain, a transmembrane domain, and a cytosolic domain. These above properties allow this unique polypeptide – heterodimeric family consisting of 1 to 18 alpha chains and 1 to 8 beta chains (currently allowing for 24 different functional integrins to occur in humans) to connect to the outside world (i.e. the world wide web of the ECM). In addition to the individual 24 identified integrins identified there exists another additional mode of communication as they can cluster together just as multiple computers can be clustered to create a "google-like" search engine to expand exponentially the cells communication skills. RO Hynes [47] has presented interesting information that there are altered states of the integrins resting on the plasma membrane: The inactive state, whereintegrins rest flaccidly upon the cell surface and an active state, where the integrin stands erect and at full attention, and thus involve the previously mentioned clustering of integrins expanding their communication skills.

This marvelous communication system of integrins allows the cell to share information and communicate bi-directionally with the ECM. In order to survive as an organism the dictum of "no cell is an island" holds true. The cell must stay "connected" either through cell-cell adhesion (connexins) or to its matrix (through integrin-matrix ligand binding sites of the ECM) or undergo apoptosis – or more specifically anoikis (self suicide) triggered by loss of contact with the ECM [46-54].

## The role of matrix metalloproteinases: MMPs and their inhibitors TIMPs

The Interstitial or fibrillar collagen types I-III are the primary collagens in the interstitial ECM. They are maintained and under the control of the family of zinc-dependent, redox sensitive, endopeptidases: matrix metalloproteinases (MMPs) (table 5). There is a delicate physiologic balance between the tearing down, rebuilding, tailoring, and sculpturing (remodeling) of the collagens within the ECM.

**Table 5:** Extracellular matrix degradation mechanisms: Focus on MMP

***Type***	***Examples***	***Location***	***Examples of Substrates***
Serine protease	Plasmin, urokinase, cathepsin G, TPA	Pericellular Extracellular	Fibrin, fibronectin, laminin, some proteoglycans
Cysteine protease	Cathepsins B, D, H, L, N, and S	Generally Cytosol-lysosomal	Collagen, elastin. & proteoglycans
MMPs: Matrix Metalloproteinases	Interstitial collagenases (MMP-1)	Extracellular	Collagens I, II, III, VII, and X
Basement Membrane	Gelatinase A (MMP-2) 72 kDa	Extracellular	Collagens IV – BM, V, VII, and X Elastinolytic
	Stromelysin-1 (MMP-3)	Extracellular	Collagens IV, III, V, and IX; laminin, fibronectin, elastin, proteoglycans
	PUMP-1 (MMP-7)	Extracellular	Gelatin, fibronectin, laminin, collagen type IV, procollagenase, and proteoglycan core protein
Of Emerging Importance! [134]	Neutrophil Elastase - collagenase (MMP-8): Activated by CD-40 ligand	Extracellular Macrophage Endothelial Cell of Vasa Vasorum	Collagens I, II, and III and proteoglycans Elastinolytic Internal Elastic Lamina. Activates MMP-2 -9
Basement Membrane	Gelatinase B (MMP-9) 92 kDa	Extracellular	Collagens IV – BM V, VII, and plus Elastinolytic
	Stromelysin-2 (MMP-10)	Extracellular	Similar to stromelysin-1
	Stromelysin-3 (MMP-11)	Extracellular	Gelatin, fibronectin, and proteoglycans
	Metalloelastase (MMP-12)	Extracellular	Elastin
Membrane type MMP	MT-MMP (MMP-14)	Cell surface	Collagen IV, gelatin, and progelatinase A
Cardiac integrin MMP	Disintegrin Metalloproteinse (DMP)	Membrane type integrin matrix degrading MMP	Endothelial Cardiac Integrin

MMPs show a wide range of specificity for various substrates, which include: native and partially degraded fibrillar collagens, basement membrane collagens, proteoglycans, elastin, and fibronectin. The ability of certain MMPs, such as MMP-2, MMP-3, MMP-9, and MMP-12, to hydrolyze elastin are of particular importance in terms of their effects on the vasculature not only within the arterial-vascular wall of vulnerable plaques but also within the vulnerable renal-mesangial stalk, which may result in plaque rupture and glomerular collapse, respectively.

MMP-2 or gelatinase A (72 kDa) and MMP-9 or gelatinase B (92 kDa) are the two enzymatic proteinases, which are primarily responsible for tearing down type IV collagen BM. These are synthesized by multiple vascular cell types including the endothelial cell and its supportive cell types: the pericyte and podocyte, VSMCs, renal mesangial cells, the fibroblast and the vascular fibroblast and myofibroblast, and the systemic-circulatory derived monocyte derived macrophage, as well as, the local tissue macrophage. The following two statements are necessary for a better understanding of their complicated roles in the ECM remodeling process.

MMP-2 may be considered to be a constitutive enzyme, while MMP-9 may be considered to be inducible in these various cell types.

The more robust the MMP signal and actions within the ECM, the more robust the repair mechanism of newly formed collagen synthesis.

There is a delicate balance between MMPs and their naturally occurring inhibitors (tissue inhibitors of matrix metalloproteinases or TIMPs). In the physiologic state the organism attempts at all times to achieve homeostasis. As a result there are checks and balances in the MMP – TIMP ratio. Additionally, it is important to understand that MMPs reside not only in the secreted form in the circulatory system, but also reside within the zymogen form within the ECM and remain in an inactive-latent or pro MMP state until they are activated by the tissue or urokinase plasminogen activator (tPA – uPA) driven plasmin. MMP-9 specifically can be activated by MMP-2 and MMP-3, as well as, membrane anchored MT1-MMP at the cell surface, converting proMMP-2 to active MMP-2 [55].



As will discussed later, the elevations of plasminogen activator inhibitor, elevated in MetS, prediabetes, and overt T2DM may have a devastating and detrimental effect on plasmin production and thus activation of latent or pro MMPs. This may play a role in matrix accumulation within the capillary BM, impaired fibrinolysis, impaired wound healing, and the impaired arteriogenesis associated with the vascular paradox.

MMP-9 has been shown to be elevated in T2DM and, in addition, the role of redox stress was shown to play an important role [56]. In addition to the endothelial cell, tissue and circulatory monocyte derived macrophage of chronic inflammation, the mesangial cell of the renal glomerular mesangium, the endothelial supporting podocyte and pericyte, and the cardiac myofibroblast each play an important role in synthesizing the inducible MMP-9. Because of the finding of an elevated MMP-9 in T2DM, both of these supportive cells may play an important role in the maintenance and the over-expression of type IV collagen in the endothelial CBM.

The integrin receptor for hyaluronan is CD-44 and it has been shown that MMP-9, in its active form, is associated with the cell surface via this CD-44 – hyaluronan integrin, which demonstrates just how connected the proteolytic MMP-9 enzyme activity is related to proteoglycans of the matrix and the cell surface membrane anchored MT1-MMP and the integrins. Also, the inactive or pro MMP-9 has a strong binding affinity via its gelatin-binding domain to bind to the alpha 2 (IV) chain of BM type IV collagen [55]. Recently, in our laboratory, we have been able to demonstrate decreased endothelial cell density with increased apoptosis of endothelial cells in the hearts of mice treated with alloxan *vs*. controls. Additionally, there was a decrease in NO and an increase in peroxynitrite and ROS in these same animals thus, linking the importance of cellular apoptosis, MMP-9 and redox stress. We then compared these findings of alloxan-induced diabetes in MMP-9 knockout mice to alloxan-induced diabetes in the wild type. Alloxan-induced diabetes MMP-9 -/- mice did not have induced apoptosis and did not have a decrease in endothelial cell density when compared to wild type alloxan-induced diabetes [57,58].

These findings may apply to the beta cell within the islet, as all cells require an integrin-matrix ligand binding for survival. The MMP-9 may also decrease the larger size amylin derived islet amyloid fibrils to the more intermediate size toxic amyloid particles and contribute to apoptosis as described by Janson *et al*. [59]

Death AK *et al*. have recently been able to demonstrate that MMP-1, MMP-2, and MMP-9 had an increased expression and activity by endothelial and monocyte derived macrophage cells under the influence of an elevated glucose in diabetic relevant concentrations. Additionally, they were able to show a decrease in MMP-3, while there was no significant effect on TIMP-1 expression. This dysregulation of MMP/TIMP system could lead to a net activation and a robust matrix degradation of type IV collagen within the basement membrane leading to a more robust laying down of new and reassembled type IV collagen as well as other BM matrix constituents [60]. This could also add to the vulnerability of vulnerable plaques, as well as, accelerating the underlying atherosclerotic process within the arterial vessel wall.

Tsilibary EC has been able to demonstrate an increase in type IV collagen, a decreased expression of MMP-2 and MMP-3, and an increased expression of TIMP-2 under high glucose conditions [34]. Their group has also been able to elegantly demonstrate a dysregulation of integrin expression, in that, under high glucose conditions the normal pattern of type IV collagen – integrin expression was shifted from alpha(3)beta(1) and alpha(2)beta(1) to a pattern of expression for alpha (v)beta(3) and alpha(5)beta(1). This alteration between the integrin – type IV interaction could certainly be playing a role in the loss of foot processes and the narrowed filtration slits of the supportive glomerular endothelial podocyte. Also, if there was robust MMP-9 production the integrin – matrix ligand binding could also become disrupted resulting in the loss of attachment of the podocyte to the ECs resulting in anoikis (apoptosis as a result of loss of attachment by integrin-matrix ligand binding sites: see previous section on integrins) [34].

## The guardian angels of the capillary endothelial cell: the pericyte and podocyte

Capillary endothelial cells are supported and nourished by the pericyte in the systemic vascular bed and by the podocyte (the renal visceral epithelial cell) of the renal glomerular vascular bed. These cells play a similar supportive role for the endothelium and may be considered to be their guardian angels.

Each of these cells is very sensitive to oxidative – redox stress and the toxicity of hyperglycemia, be it intermittent (postprandial as in prediabetes) or sustained in overt T2DM. Once the protective effect of the pericyte and podocyte are lost by dysfunction or loss by apoptosis, the capillary endothelium becomes highly vulnerable to the multiple toxicities (A-FLIGHT-U toxicities) (table 1) and injurious stimuli (figure 1) associated with the MetS, prediabetes, and overt T2DM.

The initial structural findings demonstrated by electron microscopy were the loss of the foot process between the pericyte, podocyte, and capillary endothelial cells and eventually the loss of these two supportive cells, in part, through apoptosis. The vulnerability of these two specific cells are quite reminiscent of the beta cell within the islets of the pancreas in regards to their being unable to properly handle the elevated tension of oxidative – redox stress. These two unique cells support the capillary endothelium in synthesizing and maintaining their shared tri-laminar BM consisting of a lamina rara – lamina densa – lamina rara [61-65].

## The role of advanced glycosylation endproducts (age) and ECM remodeling

AGE and the resultant cross-linking of proteins make the AGE-collagen adducts less likely to be degraded by MMPs (thus allowing for accumulation). The BM thickening associated with obesity and MetS are probably reversible, while the BM thickening associated with diabetes, hyperglycemia, ROS, and PKC activation are irreversible due to AGE formation and cross-linking.

Additionally, AGE directly quench endothelial nitric oxide and the oxidative – redox stress generated in their formation contributes to endothelial nitric oxide quenching, as well. This contributes to the endothelial cell dysfunction associated with the MetS, prediabetes, and T2DM. These affects of AGE not only contribute to the thickening of the capillary BM but also contribute to the endothelial dysfunction seen early on and contribute to its progressive deterioration as the underlying glycosylated type IV collagen accumulates within the BM [66-68]

**Figure 4:**
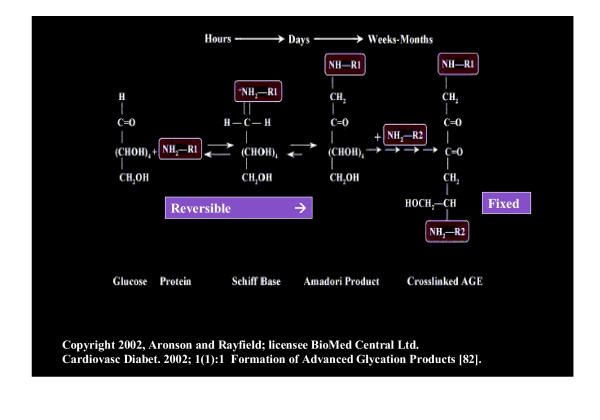
**Formation of age. **The formation of AGE, as a result of chronic hyperglycemia, is complex and this figure demonstrates the steps and time frames involved in the formation of AGE and cross-linking of proteins. This complex process is reversible until the **NH-R**^(***n***) ^'s are crosslinked.

The formation of AGE also will contribute to the accumulation of the interstitial fibrillar collagens responsible for the interstitiopathy associated with diabetic nephropathy and diabetic cardiomyopathy (figure 4).

## Remodeling of ECM in metabolic syndrome

When T2DM is clinically diagnosed there may already be diabetic complications, such as: retinopathy (20%), nephropathy (8%), neuropathy (9%), atheroscleropathy – macrovascular disease (50%), and endotheliopathy (endothelial dysfunction: approaching 100 %) [69-72]. These clinical findings have lead clinicians to the hypothesis that either impaired glucose tolerance and impaired fasting glucose have preexisted for some time prior (in the 5–10 year range) to the diagnosis of overt T2DM or that the natural history of T2DM with its origins rooted in the MetS have contributed to the preexisting diabetic complications at the time of clinical diagnosis. Other possible explanations could be that polygenic T2DM is a vascular disease rooted in endothelial genetic defects and occurs as a result of interactions with environmental stressors such as over nutrition, obesity, and under exercise in the MetS with hyperglycemia being a late manifestation [73].

**Figure 5:**
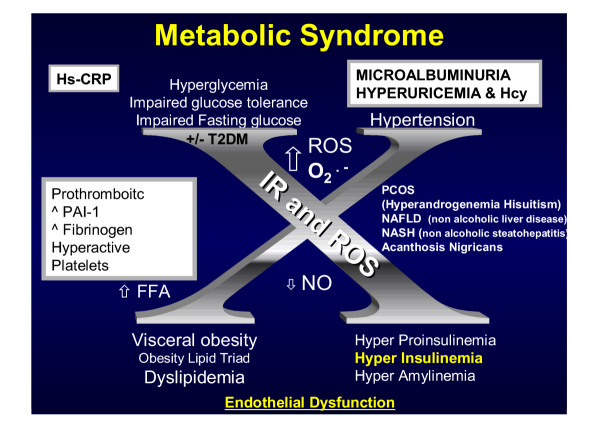
**The metabolic syndrome "Reloaded". **MetS (Syndrome X) "reloaded" is a unique clustering of clinical syndromes and metabolic derangements. Reaven initially described the MetS in 1988. He initially discussed the four major determinants consisting of: I. Hypertension. II. Hyperinsulinemia. III. Hyperlipidemia (Dyslipidemia of elevated VLDL – triglycerides, decreased HDL-cholesterol, and elevated small dense atherogenic LDL-cholesterol). IV. Hyperglycemia or impaired glucose tolerance, impaired fasting glucose, or even overt T2DM and the central importance of insulin resistance and hyperinsulinemia. The important association of polycystic ovary syndrome (PCOS), hyperuricemia, fibrinogen, hsCRP, microalbuminuria, PAI-1, and more recently reactive oxygen species (ROS), NASH, and the damaging oxidative potential of Hcy and endothelial dysfunction have all contributed to a better understanding of this complicated clustering phenomenon. ROS, and those with a white background: Hyperuricemia, microalbuminuria, hyperhomocysteinemia, highly sensitive CRP, indicate the newer additions giving rise to the new terminology: Metabolic Syndrome Reloaded.

The MetS (figure 5) consists of four major components: I. Hyperinsulinemia, II. Hypertension, III, Dyslipidemia (Lipid Triad of increased triglycerides, increased small dense LDL-cholesterol, and decreased HDL-cholesterol) – Obesity, and IV.

Hyperglycemia. The hyperglycemia – glucotoxicity section [G] of the A-FLIGHT-U toxicities are discussed elsewhere in this article and therefore the focus will be on the remaining four categories of the MetS: Volume, Pressure, Dyslipidemia – Obesity, and Hyperglycemia.

### I. Volume

Hyperinsulinemia, hyperproinsulinemia, and hyperamylinemia all three independently and synergistically activate angiotensin II and increase renal blood flow resulting in renal hyperfiltration (Section [A] amylin toxicity and Ang II toxicity and section (I) insulin toxicity of table1). This results in both increased volume and pressure. Increased volume and hyperfiltration results in dilated glomerular capillaries, expansion of Bowman's space, glomerular hypertrophy and expansion and capillary BM thickening.

### II. Pressure: hypertension

**Figure 6:**
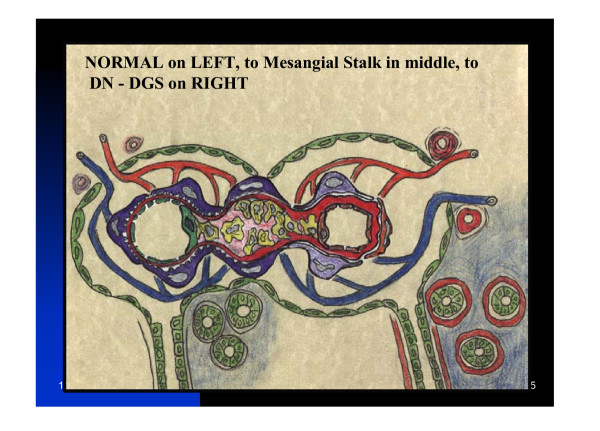
**Renal glomerular Remodeling. **This image portrays a normal nephron unit on the left transitioning to an abnormal remodeled nephron unit with changes representative of diabetic nephropathy and changes of glomerulosclerosis. Left: Normal renal capillary glomerular and tubulo-interstitial structures. Transitioning to the Center of the image is the mesangial stalk with mesangial cell hyperplasia (yellow) and mesangial expansion with loss of foot processes of the podocyte (also termed visceral epithelium) (blue) and increasing thickness of the glomerular BM (red). Right: Increased capillary glomerular BM thickening (red) with atrophic podocytes and loss of foot processes of the podocyte (blue) to the capillary glomerular endothelial cell. Right: Also depicts tubulo-interstitial fibrosis with expansion of the peritubular (blue) extracellular matrix (fibrosis) with an increased thickening of the tubular BM (red). Just below the efferent (blue) arteriole is depicted hyaline arteriolosclerosis and just above the afferent arteriole (red) is depicted hyperplastic arteriolosclerosis with its characteristic "onion skin" like changes. The thickened BMs, arteriolar changes, and the mesangial expansion all are PAS+, hyaline staining, and contain large amounts of type IV collagen with increased laminin and fibronectin with concurrent decreased amounts of heparin sulfate proteoglycan (perlecan). Continuous with the proximal tubules (green) is the outer parietal epithelial cells (green), which constitutes the outer structure of Bowman's capsule and Bowman's space.

Hypertension is part and parcel of the MetS and results in vascular remodeling consisting of arteriolosclerosis of the BM especially in the afferent arteriole of the kidney (figure 6) as well as, remodeling of type I-III collagen of the renal tubular interstitium. Additionally there is arterial intimal remodeling in thehypertensive MetS patient [74].

Hypertension is associated with oxidative stress of the arterial intima (associated with the production of ROS), which can activate protein kinase C (PKC) and transforming growth factor beta affecting both the BM of arterioles, as well as, the interstitium associated with the vascular intima and the interstitium of the renal tublular epithelium (figure 6).

### III. Dyslipidemia – obesity

Lipid peroxidation results in ROS, which may activate the PKC beta isoform resulting transforming growth factor-beta activation and glomerular BM, matrix expansion, and interstitial renal tubular remodeling.

Obesity is associated with insulin resistance (IR) and compensatory hyperinsulinemia, hyperproinsulinemia, and hyperamylinemia, which are known to activate the renin angiotensin system and Ang II. Likewise, Ang II is known to induce ROS through the membraneous reduced nicotinamide adenine dinucleotide phosphate oxidase enzyme system and transforming growth factor-beta, which results in glomerular and renal tubular interstitial remodeling. We have been able to demonstrate in an (high-fat diet) obesity dog model an increase in arterial pressure, hyperinsulinemia, activation of the renin-angiotensin system, glomerular hyperfiltration, a trend to elevation of transforming growth factor beta and structural changes including: expansion of Bowman's capsule, increased mesangial matrix and thickening of the glomerular and tubular basement membranes and the number of dividing cells in the kidney [75].

The natural progressive history of T2DM with associated MetS and IR – associated compensatory hyperinsulinemia may result in a remodeling of the ECM prior to the diagnosis of overt T2DM. The volume, pressure, dyslipidemia, and obesity can also stimulate these same mechanisms and affect the intima, BM, and interstitial collagen within the myocardium resulting in a periodic acid Shiff positive staining of arterioles, capillary BMs, and the myocardial interstitium, in addition too, the remodeling of collagens type I-III of the interstitial matrix in target organs including the myocardium [76].

**IV. *Hyperglycemia ***impaired glucose tolerance (IGT), impaired fasting glucose, and overt T2DM are discussed later in the section entitled: Central role for protein kinase C beta isoform. Hyperglycemia mechanisms.

## Remodeling of ECM in diabetic complications

Regarding macrovascular disease, Norhammer A *et al*., were able to demonstrate that 70% of patients with an acute myocardial infarction have either diabetes or IGT [77]. This elevated association of IGT and or diabetes points to atheroscleropathy and macrovascular disease. Cardiologists have noted a strong correlation of acute coronary syndromes and diabetes or IGT for some time and this study now validates their clinical suspicions. This information provides the clinician with an opportunity to possibly reverse the progressive nature of macrovascular disease in these patients by aggressive treatment through weight loss and exercise or pharmacological intervention to treat the underlying MetS state of IR and possibly the progressive beta cell dysfunction with resultant accelerated atherosclerosis and macrovascular disease. This needs to be accomplished in concert with the primary care clinician.

Regarding microvascular pathology, diabetes is the leading cause of blindness, end-stage renal disease, and a variety of debilitating neuropathies. Diabetic patients are the fastest-growing group of renal dialysis and transplant recipients, and in the USA, their 5-year survival rate is only 21 percent, which is worse than all forms of cancer combined. Over 60% of diabetic patients suffer from neuropathy, which accounts for 50% of all nontraumatic amputations in the USA [78].

## A central role for protein kinase C beta isoform (PKC)

**Figure 7:**
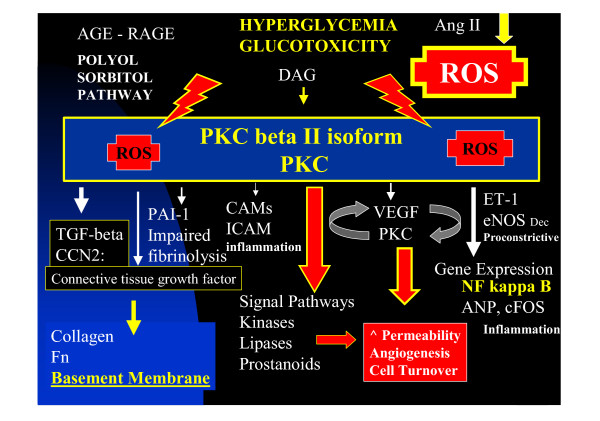
**Pkc activation. **This figure demonstrates the multiple deleterious actions and mechanisms of PKC beta II isoform on cellular function and ECM remodeling and BM thickening.

Each of the microvascular diabetic complications share a common microvascular metabolic signaling pathway through activation of PKC (figure 7) [79,80]. ROS and PKC play such important roles in each of the microvascular diabetic complications in both T1DM and T2DM. Hyperglycemia has been thought to be the stimulus for activation of PKC and the subsequent complications.

Hyperglycemia-induced mechanisms that may induce vascular dysfunction in specific sites of diabetic microvascular damage include the following:

1. Increased polyol pathway flux.

2. Altered cellular redox state with elevations of ROS.

3. Increased formation of diacylglycerol

4. Subsequent activation of specific PKC isoforms

5. Accelerated nonenzymatic formation of AGE and the AGE-RAGE connection. Activation of the receptor for AGE plays an important role.

6. Elevations of ROS.

Each of these mechanisms may contribute to the known pathophysiologic features of diabetic complications by a number of mechanisms, including the upregulation of cytokines and growth factors. Recently Brownlee M et al. has demonstrated that hyperglycemia (glucotoxicity) results in the formation of ROS, which then activate the deleterious PKC mechanism (figure 7) [81].

While the above may help to understand the increase in microvascular disease, it is felt that macrovascular disease may be an earlier occurrence and more deeply rooted in IR than from the later onset of hyperglycemia. While, at the same time a better understanding of the above numbered mechanisms play such an important role of accelerating and destabilizing atherosclerotic vulnerable plaques in the diabetic patient [82].

## Conclusion

The adaptability of the ECM and its individual components in response to an ever changing environment including its response to multiple injurious stimuli resulting in an oxidative – redox stress resulting in an excess of ROS, known to be present in MetS and T2DM, allows tissues and organs to survive. However, this adaptability – survival mechanism results in a change in form and structure resulting in fibrosis or scarring, which results in abnormal function or disease. The ECM with its communications skills enhanced through a family of cellular integrins allows for information to be exchanged in order to adapt to its ever-changing environment. This review has focused on MetS, prediabetes, T2DM, and atheroscleropathy in an effort to better understand these mechanisms in the clustering of clinical syndromes (MetS) and the specific disease state of T2DM, which are each tightly associated with the current epidemic of obesity – diabesity and a genetic predisposition of a large number of patients in order to expand our current database of knowledge.

A central theme to the injury process regarding gene activation and transcription of various factors in an attempt to respond to the multiple injurious stimuli can be related to each organ, tissue, and cell, in that, whenever there is injury the cell recapitulates its embryonic genetic memory in an attempt to heal through growth (re-growth), differentiation, development, and repair. As a result of this chronically activated wound healing mechanism, which allows for survival; we as clinicians and researchers in this field of study must constantly review and expand our knowledge in an attempt to alter the wound healing – ECM response in a manner to decrease the morbidity and mortality and the progressive nature of MetS, prediabetes, T2DM, atheroscleropathy, and their associated complications.

## List of abbreviations

AGE: advanced glycosylation endproducts

BM(s): basement membrane(s)

CBM: capillary basement membrane

ECM: extracellular matrix

GAG(s): glycosaminoglycan(s)

HSPG: heparan sulfate proteoglycans

IGT: impaired glucose tolerance

IR: insulin resistance

MetS: metabolic syndrome

MMP(s): matrix metalloproteinase(s)

PG: proteoglycan

PKC: protein kinase C

RAGE: receptor for advanced glycosylation endproducts

RGD: arginine-glycine-aspartic acid sequence

ROS: reactive oxygen species

T2DM: type 2 diabetes mellitus

TIMP: tissue inhibitor of metalloproteinase

## Competing interests

MRH, SCT, and JRS: None

## Authors' contributions

MRH conceived the idea to write this paper, MRH, SCT, and JRS collaborated to write and edit this manuscript equally.

**Table 3:** Components of the basement membrane: See Figure 2

***Component***	***Constituent Chains***	***Molecular Composition***	***Function***
Type IV Collagen:	alpha 1(IV), alpha 2(IV) alpha 3(IV) alpha 4(IV) alpha 5(IV)	Three alpha chains Structure: Polygonal shaped	Network structure Provides a structural-lattice base for the attachment of other BM macromolecules such as HSPG, laminin, enactin and Fn.
Perlecan: Heparan sulfate proteoglycan (HSPG): Proteoglycan (PG)	Polypeptide chain, side chains of GAGs	Protein Core GAG side chains Highly anionic sulfated. Structure: Multiple globular protein core with multiple polypeptide chains. See figure 2.	Electrostatic charge important for filtering. Especially in renal glomerulus.
Enactin – Nidogen: [31] Structural – Adhesive Glycoprotein	Single polypeptide chain	Structure: Dumbbell-shaped sulfated glycoprotein	Bridges Laminin and Type IV collagen. Important in assembly of the BM and changes in permselectivity properties.
Fibronectin (Fn): Structural – Adhesive Glycoprotein	Two polypeptide chains connected by two disulfide bridges.	Structural glycoprotein One of the most primitive ECM macromolecules: The first to be deposited in the embryo. Parallel to V-shaped joined by two disulfide bonds.	Connecting cells with other components of the ECM, which integrates the cell into a functional unit. Very important in wound healing.
Laminin: The most abundant glycoprotein in BMs. Structural – Adhesive Glycoprotein CABLIN: NEW Capillary Basement membrane lamina	A, B_1_, B_2_ First unique protein of the capillary basement membrane	One A and two B chains. Structure: Cruciform shape Rod like structure found only in the lamina rara of capillaries	Cell attachment Assembly of the BM Stabilization of type IV Collagen Cell-matrix attachment providing stability to the basement membrane

See Figure 2

**Table 6:** The positve protective effects of Endothelial Nitric Oxide Synthase (Enos) and Endothelial Nitric Oxide (eNO)

**The positve protective effects of eNOS – eNO**	
1.	Promotes vasodilatation of vascular smooth muscle.
2.	Counteracts smooth muscle cell proliferation.
3.	Decreases platelet adhesiveness.
4.	Decreases adhesiveness of the endothelial layer to WBCs (monocytes). Thus, the .... "Teflon effect".
5.	Anti- inflammatory.
6.	Anti- oxidant. It scavenges reactive oxygen species, locally. Acts as a chain – breaking antioxidant to scavenge ROS.
7.	Anti- fibrotic. When NO is normal or elevated MMPs are low and conversely if NO is low MMPs are elevated and active. MMPs are redox sensitive.
8.	NO has diverse anti-atherosclerotic actions on the arterial vessel wall: including antioxidant effects by direct scavenging of ROS – RNS acting as chain breaking antioxidants and anti-inflammatory effects
